# The prevalence of glucose-6-phosphate dehydrogenase deficiency in Gambian school children

**DOI:** 10.1186/1475-2875-13-148

**Published:** 2014-04-17

**Authors:** Joseph Okebe, Alfred Amambua-Ngwa, Jason Parr, Sei Nishimura, Melissa Daswani, Ebako N Takem, Muna Affara, Serign J Ceesay, Davis Nwakanma, Umberto D’Alessandro

**Affiliations:** 1 Medical Research Council Unit, Atlantic Boulevard, Fajara, The Gambia; 2 University of Manchester, Manchester, UK; 3 London school of Hygiene and Tropical Medicine, London, UK; 4 Institute of Tropical Medicine, Antwerp, Belgium

**Keywords:** Glucose-6-phosphate dehydrogenase deficiency, Malaria, Genotype, Phenotype, Prevalence

## Abstract

**Background:**

Primaquine, the only available drug effective against *Plasmodium falciparum* sexual stages, induces also a dose-dependent haemolysis, especially in glucose-6-phosphate dehydrogenase deficient (G6PDd) individuals. Therefore, it is important to determine the prevalence of this deficiency in areas that would potentially benefit from its use. The prevalence of G6PD deficiency by genotype and enzyme activity was determined in healthy school children in The Gambia.

**Methods:**

Blood samples from primary school children collected during a dry season malaria survey were screened for G6PDd and malaria infection. Genotypes for allele mutations reported in the country; 376, 202A-, 968A- and 542 were analysed while enzyme activity (phenotype) was assayed using a semi-quantitative commercial test kit. Enzyme activity values were fitted in a finite mixture model to determine the distribution and calculate a cut-off for deficiency. The association between genotype and phenotype for boys and girls as well as the association between mutant genotype and deficient phenotype was analysed.

**Results:**

Samples from 1,437 children; 51% boys were analysed. The prevalence of *P. falciparum* malaria infection was 14%. The prevalence of the 202A-, 968 and 542 mutations was 1.8%, 2.1% and 1.0%, respectively, and higher in boys than in girls. The prevalence of G6PDd phenotype was 6.4% (92/1,437), 7.8% (57/728) in boys and 4.9% (35/709) in girls with significantly higher odds in the former (OR 1.64, 95% CI 1.05, 2.53, p = 0.026). The deficient phenotype was associated with reduced odds of malaria infection (OR 0.77, 95% CI 0.36, 1.62, p = 0.49).

**Conclusions:**

There is a weak association between genotype and phenotype estimates of G6PDd prevalence. The phenotype expression of deficiency represents combinations of mutant alleles rather than specific mutations. Genotype studies in individuals with a deficient phenotype would help identify alleles responsible for haemolysis.

## Background

The reduction in the malaria burden observed in several endemic countries has revived optimism about the possible elimination of this disease [1-3]. In this context, the prospect of a massive scale up of control interventions, including the use of drugs active against sexual stages of *Plasmodium falciparum* with the aim of interrupting transmission, is of particular interest [4]. Currently, primaquine, an 8-aminoquinoline, is the only available medicine with gametocytocidal properties and its administration with artemisinin-based combination therapy (ACT) is recommended in areas where malaria elimination is considered feasible [5]. Nevertheless, primaquine may cause haemolytic anaemia in individuals with glucose-6-phosphate dehydrogenase deficiency (G6PDd), a major bottleneck for its large-scale use [6,7]. Therefore, the decision to deploy primaquine should be based on the local prevalence of G6PDd and on the evaluation of the potential risks of adverse drug reactions.

G6PDd is considered the most common enzyme deficiency, affecting about 5% of the world’s population, though in most cases it is clinically silent [8]. It is caused by mutations in the coding sequence of the gene located on the X- chromosome and thus affects mainly males when hemizygous, while females can be either homo- or heterozygous for this deficiency. G6PDd protects against severe malaria and there is a significant geographical overlap with malaria endemic areas [9,10]. Therefore, the use of primaquine represents an ethical challenge as those who could benefit the most may also be at greatest risk of harm [11]. Information on the prevalence of G6PDd is fragmented; in sub-Saharan Africa (sSA), prevalence figures have been generated using different methods so that it is difficult to compare and summarize the available information [10,12-16]. In addition, as detection methods have improved over the years, some G6PDd prevalence figures may need to be reviewed.

G6PD status is determined by molecular and/or biochemical methods [17]. Screening tests involve either quantitative or qualitative measurements of enzyme activity but are affected by several factors [18] and have reduced sensitivity in detecting heterozygous females [19]. Molecular methods provide more consistent results but are costly. Besides the wild type (G6PD B), the G6PD A variant is considered the most prevalent and the most studied in sSA [20]. This G6PD A variant is polymorphic, characterized by amino acid replacements that include the G6PD A (376G) mutation, A-(202A), A-(968C) and A-(680T). Other variants have been reported from studies of preselected population [10] and appear to be more clustered, with even higher prevalence than the classic 202A- [12], highlighting the need for a better description of the burden of this enzyme deficiency.

This study presents an update on G6PD genotype frequencies reported in The Gambia and describes the association between genotypes and phenotypes (enzyme activity) in healthy school children.

## Methods

As part of a malariometric survey [21], blood samples from children aged 5 to 14 years were analyzed for both specific G6PD mutations (genotype) and enzyme activity (phenotype). Briefly, clinically healthy children were randomly selected from 32 lower basic schools in the Upper River Region of The Gambia. After a clinical assessment, a finger prick blood sample was collected onto a Whatman filter paper for G6PD assay and parasite detection by polymerase chain reaction (PCR). Haemoglobin was measured using a portable haemoglobin analyser (Hemocue, Angelholm, Sweden). Children with a history of fever or body temperature ≥37.5°C were screened for malaria using a rapid antigen test kit (ICT Diagnostics, South Africa) and if positive, treated with artemether-lumefantrine. The filter papers were air dried and stored at 4°C until processed. The Gambia Government/MRC joint ethics committee and the national education authority approved the study. An informed consent was obtained from parents/ caregivers of selected children.

### Measuring G6PD enzyme activity

Enzyme activity was measured using a commercial quantitative ELISA kit (Atlas Medical®). It is based on the spectrophotometric measurement of reduced nicotinamide adenine dinucleotide phosphate (NADPH) released during G6PD enzyme activity and, colorimetrically by reduction of tetrazolium salt (550 nm). The readings from the 340 nm measurement were used in the calculations. A 6 mm diameter disc was punched from each dried blood spot (DBS) into a U bottom microtitre plate and eluted with 75 μL of elution agent. NADPH was measured as absorbance at 340 nm [22] in kinetic mode for 15 minutes. Enzyme activity was normalized against the protein content of blood spot eluents determined by measuring absorbance of haemoglobin at 405 nm and expressed as the normalized increase in OD per minute (slope) using the following equation: (∆OD_340nm_/∆time)/OD_405nm_.

### G6PD genotyping

DNA was extracted using protocols for the QiaExtractogen liquid handling robot (QiaGen). Allele polymorphisms were detected by real-time PCR with TaqMan® SNP genotyping assays using ABI PRISM SDS 7500 (Applied Biosystems, Foster City, CA). Oligonucleotide primers and probe genotyping assay mixes for nucleotides A376G, G202A, A542T and T968C, were also obtained from Applied Biosystems. Genotyping assay were performed in a final reaction of 25 μL, containing 12.5 μL of 2× TaqMan Universal Master Mix, 0.5 μL of 40× TaqMan SNP genotyping assay mix and at least 10ηg of genomic DNA in 11 μL of distilled water. The amplification conditions were 2 minutes at 50°C for AmpErase uracil-N-glycosylase activity and 10 minutes at 95°C for Amplitaq Gold activation, followed by 40 cycles of 15 seconds at 95°C for denaturation and 1 min at 60°C for annealing and extension. The samples were run together with the non-template control. Allelic discrimination was performed on fluorescence data using the ABI Prism 7500 allelic discrimination software and the free TaqMan-Genotyper Software (Applied Biosystems, Foster City, CA). Each sample result was verified by examining the PCR curve generated to eliminate false-positive results due to aberrant light emission.

### Analysis

Children were classified on the basis of allelic discrimination patterns as normal, mutant (hemi- or homozygous) or heterozygous (females only) while genotypes were defined for each of the polymorphic A variants; 376, 202, 968, and for the 542 variant. The distribution of G6PD enzyme activity in the sample was analysed using a two-component finite mixture model. Using the population with the higher mean activity as reference, the cut-off for activity in the sample was defined as < −2SD below the mean of this subpopulation. The association between G6PD activity and allele mutation in males and females was examined using Pearson χ^2^. A logistic regression model was used to determine the relationship between allele mutation and a deficient phenotype and the association between haemoglobin, gender and parasitaemia on deficient phenotype. Data was analysed using Stata 12 (College Station Tx).

## Results

Blood samples from 1,437 children; 728 (50.7%) of them boys were analysed. The mean age was 10 years (SD 2.3) and mean haemoglobin was 12.4 g/dl (SD 1.3), with no difference between males and females. The prevalence of malaria infection (all *P. falciparum*) determined by PCR was 14% [21]. Overall, 717 (49.9%) children did not have any mutant alleles and were classified as wild type while 162 (11.3%) were either heterozygous or homo/hemizygous for the typed variants (Table 1). Among the remaining 558 children, a single mutation was found in 522 of them, 32 had double and four triple mutations (Table 2).

**Table 1:** Prevalence of mutant alleles by gender

**Allele type**	**Female (%) N = 709**	**Male (%) N = 728**
Wild	281 (39.6)	436 (59.9)
202A-(H)	50 (7.1)	-
202A-(M)	2 (0.3)	24 (3.3)
968 (H)	24 (3.4)	-
968 (M)	4 (0.6)	26 (3.6)
542 (H)	18 (2.5)	-
542 (M)	2 (0.3)	12 (1.7)
Others*	328 (46.3)	230 (31.6)

M = Mutant, H = Heterozygote.

*Including single, double and triple mutations (see Table 2).

**Table 2:** Prevalence of other observed alleles by gender

	**Allele type**	**Female (%) N = 328**	**Male (%) N = 230**
Single	542H	2 (0.6)	-
	968H	1 (0.3)	-
	202H	188 (57.3)	-
	376H	9 (2.7)	-
	202M	90 (27.4)	215 (93.5)
	376M	2 (0.6)	7 (3.0)
	968M	-	5 (2.2)
	542M	-	3 (1.3)
Double	202H/542M	3 (0.9)	**-**
	202H/376M	1 (0.3)	**-**
	202M/542H	9 (2.7)	**-**
	202M/968H	4 (1.2)	**-**
	202M/376H	15 (4.6)	**-**
Triple	202H/376H/542H	2 (0.6)	**-**
	202M/376H/542H	2 (0.6)	**-**

M = Mutant, H = Heterozygote.

The prevalence of the 202A-, 968 and 542 mutations was 1.8%, 2.1% and 1.0%, respectively, and higher in boys than in girls (Table 1).

**Figure 1:**
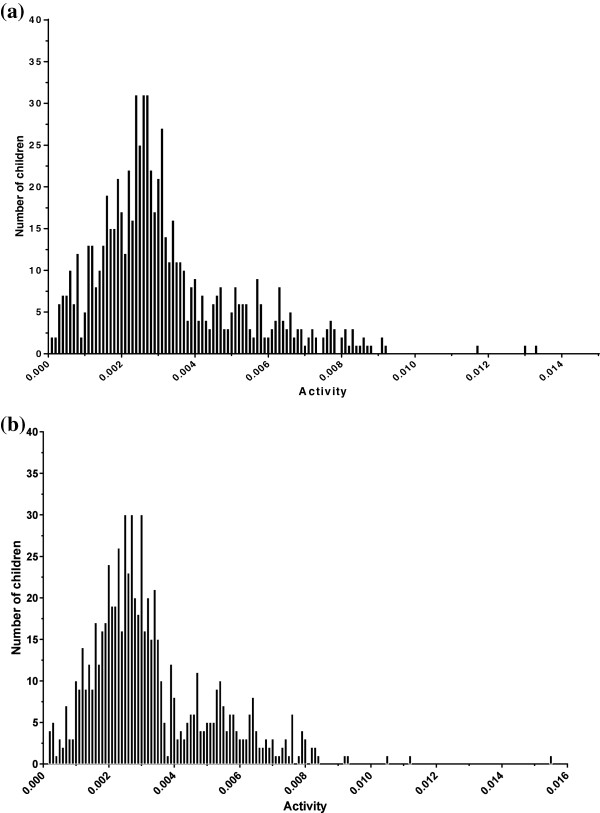
Distribution of G6PD activity in boys (a) and girls (b).

The median G6PD activity was 0.0028 (IQR 0.0020, 0.0040) and did not vary by gender however, the distribution in females showed two peaks of activity (Figures 1a and b). From the analysis, a two-component model provided the best fit for the data and the threshold for normal activity was estimated at 0.0010. The estimated prevalence of the deficient phenotype was 6.4% (92/1437), with higher frequency in boys (7.8%, 57/728) than in girls (4.9%, 35/709) with an increased odds of a deficient phenotype in boys (OR 1.64, 95% CI 1.05, 2.53, p = 0.026). The odds of a malaria infection was not significantly lower in children with a deficient phenotype (OR 0.77, 95% CI 0.36, 1.62, p = 0.49). The prevalence of the deficient phenotype was higher in boys with any of the studied deficient genotypes and in girls with a 968 or with a 542 mutations (hetero and homozygous) (Table 3). In boys, the odds of having a deficient phenotype were significantly associated with any of the mutations (Table 4). The odds of a deficient phenotype remained higher in boys after adjustment for the different genotypes studied though the strength of this association was weak (OR 1.40, 95%CI 0.86, 2.28, p = 0.175).

**Table 3:** Prevalence of the deficient phenotype by genotype and gender

	**Deficient phenotype**	
**Genotype**	**Females %(n/N)**	**Males %(n/N)**
Wild	5.0 (14/281)	6.2 (27/436)
A-(H)	6.0 (3/50)	-
A-(M)	0.0 (0/2)	16.7 (4/24)
968 (H)	4.2 (1/24)	-
968 (M)	25.0 (1/4)	30.8 (8/26)
542 (H)	11.1 (2/18)	-
542 (M)	50.0 (1/2)	25.0 (3/12)
Others	4.0 (13/328)	6.5 (15/230)

**Table 4:** Odds ratios (95% CI) of a deficient phenotype by allele mutations and gender

	**Females (709)**		**Males (728)**	
**Genotype**	**OR**	**P**	**OR**	**P**
Wild	1		1	
A-(H)	1.22 (0.34, 4.40)	0.76	-	-
A-(M)	-	-	3.03 (0.97, 9.49)	0.057
968 (H)	0.83 (0.10, 6.59)	0.86	-	-
968 (M)	6.36 (0.63, 65.08)	0.12	6.73 (2.68, 16.88)	0.00
542 (H)	2.38 (0.49, 11.4)	0.28	-	-
542 (M)	-	-	5.05 (1.29, 19.74)	0.02

## Discussion

The use of primaquine in malaria endemic areas should be balanced by the potential risk of haemolysis in G6PDd individuals hence the need for accurate estimates of the prevalence of deficiency.

The 202A- variant is considered the most frequent in sSA so that the prevalence of G6PDd is often measured by detecting this mutation. Nevertheless, in this area the 968A- variant was more frequent than the 202A- variant, confirming previous reports [10,12] and indicating that allele mutations related to G6PDd status may show distinct sub-regional distribution. A range of factors including clinical status, medication and undiagnosed haemoglobinopathies affects G6PD activity [6,23]. Children attending school were chosen in this study because they were considered clinically healthy at the time of sampling. The prevalence of malaria infection was 14%, a relatively substantial value when considering that the survey was carried out at the end of the dry season, after several months of virtually no transmission.

G6PDd has been associated with a protective effect against severe malaria [24] in males but not in females [25] and has been associated with multiple mutations rather than a specific mutant genotype [10]. However, its effect on asymptomatic infection is less clear. In this study, G6PDd showed a weak association with asymptomatic infection and this is similar to observations in other studies assessing the effect of haemoglobinopathies, including G6PDd [26,27].

Measuring single genotypes [28-30] does underestimate the true G6PDd prevalence because it targets only the individual genotypes known to be highly frequent in the study population. From this study, about 39% of the children had a range of single or multiple allele mutations that did not fit any of the combinations that characterize the studied genotypes. These mutations cause qualitative or quantitative changes in enzyme activity that range from no to increased activity and would explain the varied clinical responses in individuals exposed to potentially haemolytic agents. There is some evidence that even where enzyme levels are low, they are never completely absent [31].

Two peaks were seen in the distribution of activity in females but not in males, which has been described with the more sensitive cytochemical assays [17,32]. The wide distribution reflects the mosaicism in normal and deficient cells and may not be fully explained by heterozygosity alone. This effect may be due to cells with mutations that cause increased activity [20] or other untyped mutations [32]. Cytochemical assays on the other hand are more reliable but more expensive.

For mass screening campaigns, qualitative testing is a feasible option as genotype methods are unlikely to be used routinely. However, newer methods, potentially adaptable for high throughput screening and addressing the limitation in females should be considered.

## Conclusion

The prevalence of G6PD deficiency and the risk of primaquine-induced haemolysis are important for deciding on the use of primaquine. The prevalence of G6PDd determined by measuring the phenotype (enzyme activity) is higher than that by genotype (mutations) analysis, though a substantial proportion of children are positive for both methods. Phenotype-based screening should be encouraged while genotype testing could be used to investigate subjects developing haemolysis but not identified as G6PDd by the qualitative tests.

## Abbreviations

ACT: Artemisinin-based combination therapy; DBS: Dried blood spot; DNA: Deoxyribonucleic acid; G6PD: Glucose-6-phosphate dehydrogenase; G6PDd: Glucose-6-phosphate dehydrogenase deficiency; MRC: Medical Research Council Unit; NADPH: Nicotinamide adenine dinucleotide phosphate; PCR: Polymerase chain reaction; sSA: Sub-Saharan Africa.

## Competing interests

All authors declare that they have no competing interest.

## Authors’ contributions

UDA and JO conceived and designed the project; JO lead the field work, analysed the data and wrote the draft manuscript. ENT designed the main study and supervised field work. SN, JP and MD processed the samples supervised by AAN, MA and DN. SJC contributed to the study design and supervised the field work; AAN analysed the G6PD sample data and reviewed the manuscript. UDA revised the manuscript text. All authors reviewed and approved the final manuscript.
